# The Impact of Artificially Sweetened Drinks on Metformin Efficacy

**DOI:** 10.3390/nu17050797

**Published:** 2025-02-26

**Authors:** Esraa Ismail, Xiaofei Chi, Mallika Bhatta, Jennifer Hosford, Angelina Bernier

**Affiliations:** 1 College of Medicine, University of Florida, Gainesville, FL 32610, USA; israa88_isma3el@yahoo.com (E.I.); mbhatta@ufl.edu (M.B.); 2 Division of Pediatric Endocrinology, UF Health, Department of Pediatrics, University of Florida, Gainesville, FL 32608, USA; 3 Division of Pediatrics Research Hub (PoRCH), UF Health Department of Pediatrics, University of Florida, Gainesville, FL 32610, USA; xiaofei.chi@peds.ufl.edu (X.C.); jennifer.hosford@peds.ufl.edu (J.H.)

**Keywords:** metformin, non-nutritive sweeteners, prediabetes, obesity

## Abstract

Background: Artificially sweetened beverages (ASBs) are commonly recommended as a substitute for sugar-sweetened beverages (SSBs) in dietary counseling. Childhood obesity, associated with comorbidities like type 2 diabetes (T2D), has risen alongside increased consumption of both SSBs and ASBs. Metformin, a common treatment for pediatric T2D, affects GDF-15, a hormone involved in weight regulation. This pilot study examines the impact of ASBs on the therapeutic effects of metformin in pediatric patients with obesity and prediabetes, focusing on growth differentiation factor 15 (GDF-15) as a potential mediator. Methods: Forty-six children aged 10–21 years were randomized into two groups: one consuming non-sweetened beverages (USB) and the other consuming ASBs during a 12-week metformin intervention. Results: While the USB group showed a greater decrease in the point estimate for mean BMI (−0.55 ± 1.49 USB vs. −0.23 ± 1.60 ASB) and an increase in the point estimate for mean GDF-15 (33.40 ± 58.34 in USB vs. 19.77 ± 85.87 in ASB), these differences were not statistically significant (*p* = 0.6). As a hypothesis-generating exercise, change in insulin resistance was explored. While again lacking statistical power, we observed that more participants in the USB group showed improvements in insulin resistance. Conclusions: Additional studies are needed to fully elucidate the impact of non-nutritive sweeteners on metabolic health and treatment outcomes in pediatric obesity.

## 1. Introduction

The global obesity epidemic is a significant public health concern, with childhood obesity affecting approximately one in five children in the United States. The prevalence of comorbidities, such as prediabetes and type 2 diabetes (T2D), is also rising in children [1,2]. Childhood obesity is particularly alarming due to the increased risk of adult obesity and associated non-communicable diseases. Even when excess weight is lost in adulthood, childhood obesity is linked to worse long-term morbidity and mortality outcomes [3,4].

The rise in obesity and its associated complications has paralleled the increased consumption of sugar-sweetened beverages (SSBs), which typically utilize added sucrose, high-fructose corn syrup, or fruit juice concentrates [5]. While the causality remains unclear, the association between SSB consumption and obesity is well established [6]. Additionally, increased intake of SSBs has been linked to insulin resistance, metabolic syndrome, T2D, and cardiovascular morbidity, starting at early ages [7,8].

Non-nutritive sweeteners (NNS), marketed as healthier alternatives to SSBs due to their low or zero-calorie content, have seen a significant rise in consumption in recent years [9]. Initially believed to be metabolically inert, NNS are now associated with adverse effects, including impaired glucose tolerance, weight gain, and worsening cardiovascular outcomes. These negative effects may stem from alterations in the gut microbiome and long-term disruption of the neurohormonal control of satiety [10,11,12].

Metformin, a widely used biguanide drug, has been employed for nearly 30 years due to its safety and low cost. FDA-approved as a first-line treatment for pediatric T2D in children as young as 10, metformin improves the metabolic complications of obesity and contributes to a moderate weight loss, though its effects are not always consistent [13,14]. Metformin’s mechanisms involve the activation of AMP-activated protein kinase, and, more recently, growth differentiation factor 15 (GDF-15) has been identified as a biomarker for metformin’s therapeutic effects [15].

GDF-15 is a peptide hormone produced in several tissues, including the liver, kidneys, and intestines, in response to stress. It regulates food intake, energy expenditure, and body weight by interacting with the GDNF receptor alpha-like (GFRAL) in the brainstem, promoting weight loss [16]. Metformin has been shown to increase GDF-15 release, primarily from the gut. This increase in GDF-15 is associated with improved body weight regulation, appetite, and insulin sensitivity, although some studies suggest that glucose-lowering effects may occur even without a rise in GDF-15 [17,18,19].

Dietary composition, particularly sweeteners, can influence the therapeutic effects of medications [20]. A recent study in mice has shown that consuming non-nutritive sweetened beverages (e.g., saccharin) impairs the therapeutic effects of metformin on glucose control and weight loss, while also reducing the metformin-induced GDF-15 release compared to sugar-sweetened or water beverages [21]. This pilot study explores whether a similar effect occurs in pediatric clinical practice, hypothesizing that non-nutritive sweetened drinks may impair metformin-induced satiation, weight loss, and glucose tolerance, with GDF-15 mediating this effect.

## 2. Materials and Methods

Participants were recruited from the UF Pediatric Obesity & Metabolic Clinic in Gainesville, with the goal of recruiting 40 patients. Sample size estimation was based on guidelines for pilot studies, focusing on feasibility, acceptability, and variability to inform a future efficacy trial. Anticipating a 20% dropout, 20 participants were enrolled per group (N = 16 per group post-dropout).

Preclinical screening and chart reviews were performed to identify eligible participants, who were contacted for appointments and asked to fast. Inclusion criteria included children aged 10–21 years with a diagnosis of obesity (BMI ≥ 95th percentile or ≥30 kg/m^2^) and prediabetes (A1c 5.7–6.4%). Exclusion criteria included prior oral hypoglycemic treatment, hypertension (SBP > 180 mmHg or DBP > 100 mmHg), and self-reported pregnancy. After consent, participants were randomized into a two-arm, 12-week, open-label metformin (500 mg BID) intervention: one group was advised to avoid all sweetened drinks, while the other was allowed to consume artificially sweetened beverages (ASBs). All participants were instructed to consume at least three drinks per day, with handouts detailing the recommended options. Computer-generated block randomization was used to allocate participants to different treatment groups in a block design with increments of four. Participants were randomly assigned to different treatments within each block.

Upon recruitment, participants underwent comprehensive assessments of physical health, eating behaviors, and laboratory tests (fasting glucose, insulin, A1c, and GDF-15). Anthropometric measurements (height, weight, and BMI) were also recorded. Beverage intake was assessed using a modified Beverage Frequency Questionnaire (BFQ) based on validated instruments for children and young adults (16–30 years). We combined items from two validated questionnaires and edited them in a limited fashion to make the distinction between unsweetened and artificially sweetened drinks while adding several examples to each category to help improve accurate selection (see Supplementary Materials S1). Bi-weekly assessments were collected electronically via email and stored in REDCap.

At the end of the 12-week intervention, participants returned for another fasting assessment and lab draws. Additional data on hunger, eating behavior, and metformin compliance were collected (see Supplementary Materials S2).

## 3. Statistical Analysis

Data were summarized as frequencies and percentages for categorical variables and as means with standard deviations (SD) for continuous variables. Fisher’s exact test was used for categorical variables, and paired *t*-tests were applied to assess changes from baseline between the groups. Statistical significance was set at alpha ≤ 0.05, with two-sided hypothesis tests. Analyses were conducted using SAS version 9.4 (Cary, NC, USA).

## 4. Results

Among the 46 enrolled participants, 25 (54.3%) were female, 25 (54.3%) were Black/African American, and 41 (89.1%) were non-Hispanic or non-Latino. At baseline, 14 participants (30.4%) reported occasionally feeling hungry, 21 (45.7%) sometimes asked for a second serving after meals, and 40 (87%) snacked between meals (Table 1).

**Figure 1:**
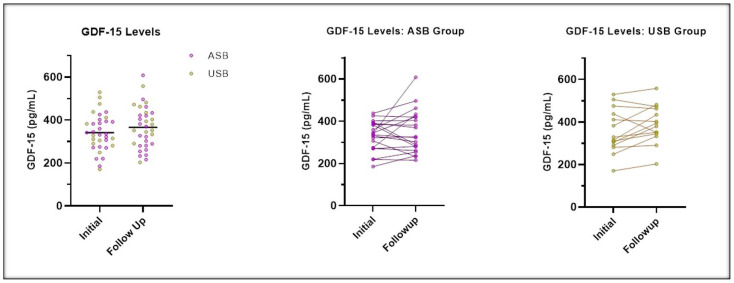
Changes from baseline to the follow-up at 12 weeks for GDF-15 levels.

Thirty-six participants (44% from the USB group) completed the study. Table 2 summarizes changes in BMI, BMI 95th percentile, BMI z-score, and GDF-15 from baseline to the 12-week follow-up. Although the USB group showed a greater decrease in BMI, %95th percentile, and BMI z-score, and a greater increase in GDF-15 levels (Figure 1) compared to the ASB group, none of these changes were statistically significant. There was no change in A1c or dietary intake.

As an exploratory and post-hoc analysis, a binary categorization of the changes showed that nine out of 10 children in the USB group had a decrease in HOMA-IR, compared to seven out of 19 in the ASB group (*p* = 0.0084). No significant differences were observed for BMI, BMI 95th percentile, BMI z-score, HbA1c, or GDF-15 (Table 3).

## 5. Discussion

Metformin has been shown to improve weight management and glucose control in children, and its effects are linked to changes in GDF-15. Metformin is also known to positively affect gut microbiome [22] and this could be altered or attenuated with the use of NNS. This pilot study investigates the impact of ASB consumption on the therapeutic effects of metformin in pediatric patients, with a focus on GDF-15 as a potential mediator. Point estimates for reduction in BMI and increase in GDF-15 were higher in the USB arm versus the ASB arm but did not reach statistical significance. No changes were observed in A1c or dietary intake, and metformin compliance was similar across groups. As a hypothesis-generating post-hoc analysis, we explored the binary relationship among differences in BMI, GDF-15, and HOMA-IR and found that a greater number of subjects in the USB arm versus the ASB arm experienced a decrease in HOMA-IR.

The long-term health effects of NNS, particularly in relation to addressing childhood obesity and its comorbidities, are unclear. Observational studies have provided conflicting results regarding the association between NNS consumption and obesity or T2D risk [21]. While the mechanism is not clear, they suggest that this could be related to alterations in the gut microbiome following dietary changes and metformin treatment. A recent mouse study indicates that NNS impairs metformin’s therapeutic effects, including a decrease in GDF-15 release [23]. This suggests that NNS may alter the metabolic response to metformin, potentially through gut microbiome modulation, which has also been shown to influence GDF-15 release [24,25].

Notably, this was the first study to explore the effects of NNS on metformin-induced GDF-15 responses in obese pediatric patients. Given the growing evidence of the potential harmful effects of NNS and the need to address childhood obesity and its comorbidities, further detailed explorations of the pathophysiology and mechanistic impacts of NNS on metabolic health are needed. Despite the novelty of this work, we recognize the many limitations of this pilot study. First and foremost, the small sample size and the lack of statistical significance limit our ability to make any conclusive statements about the potential interaction between metformin and NNS. That said, we also acknowledge that a larger, more definitive study, may, in fact, confirm the lack of statistical significance seen in this pilot (REF from the reviewer here). In addition, we note that self-reported diet and beverage consumption can be inaccurate or influenced by memory recall. In the case of this small pilot, we chose the short 24 h recall to address recall bias and, in order to improve accuracy, provided education to the participants about accurate food reporting. Additionally, we used a specific beverage frequency questionnaire for collecting details to enhance data accuracy.

That said, this was the first study to explore metformin-responsive GDF-15 secretion in a pediatric population with prediabetes and obesity. The intention-to-treat population for pediatric obesity included a higher percentage of African American participants, reflecting the increased risk of obesity within this demographic. Larger, longer-duration studies are necessary to confirm these findings and further investigate the underlying mechanistic pathways.

## 6. Conclusions

In this pilot study, we observed no statistical differences in BMI or GDF-15 levels in the USB group compared to the ASB group. The limited sample size and short intervention duration may have hindered our ability to detect significant differences. Further research with larger samples and extended follow-up periods is needed to clarify the effects of NNS on metformin’s therapeutic outcomes in pediatric obesity.

## Figures and Tables

**Table 1:** Baseline characteristics. Unsweetened beverage group (USB); artificially sweetened beverage group (ASB).

	All Participants				Completers with Follow-Up Data at 12 Weeks			
	Overall (N = 46)	USB (N = 23)	ASB (N = 23)	*p*-Value	Overall (N = 36)	USB (N = 16)	ASB (N = 20)	*p*-Value
Sex at birth (N,%)				1.0000				0.7486
Male	21 (45.7%)	11 (47.8%)	10 (43.5%)		19 (52.8%)	9 (56.3%)	10 (50.0%)	
Female	25 (54.3%)	12 (52.2%)	13 (56.5%)		17 (47.2%)	7 (43.8%)	10 (50.0%)	
Race (N,%)				0.2942				0.2155
White/Caucasian	16 (34.8%)	8 (34.8%)	8 (34.8%)		11 (30.6%)	5 (31.3%)	6 (30.0%)	
Black/African American	25 (54.3%)	11 (47.8%)	14 (60.9%)		22 (61.1%)	8 (50.0%)	14 (70.0%)	
Native Hawaiian or Pacific Islander	1 (2.2%)	0 (0%)	1 (4.3%)		0 (0%)	0 (0%)	0 (0%)	
More than one race	2 (4.3%)	2 (8.7%)	0 (0%)		1 (2.8%)	1 (6.3%)	0 (0%)	
Unknown	2 (4.3%)	2 (8.7%)	0 (0%)		2 (5.6%)	2 (12.5%)	0 (0%)	
Ethnicity (N,%)				1.0000				0.6371
Hispanic or Latino	5 (10.9%)	3 (13%)	2 (8.7%)		5 (13.9%)	3 (18.8%)	2 (10.0%)	
Non-Hispanic or Non-Latino	41 (89.1%)	20 (87%)	21 (91.3%)		31 (86.1%)	13 (81.3%)	18 (90.0%)	
How often do you feel hungry? (N,%)				0.5218				0.4045
Never	2 (4.3%)	0 (0%)	2 (8.7%)		1 (2.8%)	0 (0%)	1 (5.0%)	
Rarely	5 (10.9%)	3 (13%)	2 (8.7%)		3 (8.3%)	2 (12.5%)	1 (5.0%)	
Sometimes	14 (30.4%)	6 (26.1%)	8 (34.8%)		10 (27.8%)	3 (18.8%)	7 (35.0%)	
Most of the time	18 (39.1%)	9 (39.1%)	9 (39.1%)		15 (41.7%)	6 (37.5%)	9 (45.0%)	
All the time	7 (15.2%)	5 (21.7%)	2 (8.7%)		7 (19.4%)	5 (31.3%)	2 (10.0%)	
Do you ask for ‘seconds’ after meals? (N,%)				0.3514				0.1325
Never	4 (8.7%)	3 (13%)	1 (4.3%)		2 (5.6%)	2 (12.5%)	0 (0%)	
Rarely	7 (15.2%)	5 (21.7%)	2 (8.7%)		4 (11.1%)	3 (18.8%)	1 (5.0%)	
Sometimes	21 (45.7%)	10 (43.5%)	11 (47.8%)		17 (47.2%)	7 (43.8%)	10 (50.0%)	
Most of the time	11 (23.9%)	3 (13%)	8 (34.8%)		10 (27.8%)	2 (12.5%)	8 (40.0%)	
All the time	3 (6.5%)	2 (8.7%)	1 (4.3%)		3 (8.3%)	2 (12.5%)	1 (5.0%)	
Do you get snacks in between meals or in the evening? (N,%)				0.6652				0.6722
No	6 (13%)	2 (8.7%)	4 (17.4%)		6 (16.7%)	2 (12.5%)	4 (20.0%)	
Yes	40 (87%)	21 (91.3%)	19 (82.6%)		30 (83.3%)	14 (87.5%)	16 (80.0%)	
Age (years) (mean ± SD)	13.5 ± 2.2	13.5 ± 2.4	13.6 ± 2.1	0.8969	13.2 ± 2.0	13.0 ± 1.9	13.4 ± 2.1	0.5645
BMI (mean ± SD)	40.5 ± 8.5	39.8 ± 6.7	41.1 ± 10.1	0.6146	39.7 ± 7.8	39.4 ± 6.9	39.9 ± 8.6	0.8527
% of 95th BMI percentile (mean ± SD)	152.5 ± 29.0	151.4 ± 24.5	153.7 ± 33.5	0.7910	151.6 ± 27.1	152.4 ± 24.1	150.9 ± 29.9	0.8686
BMI z score (mean ± SD)	3.2 ± 1.0	3.2 ± 0.9	3.3 ± 1.2	0.7699	3.2 ± 1.0	3.2 ± 0.9	3.2 ± 1.0	0.8841
Fasting glucose (mg/dL)	98.5 ± 14.7	94.6 ± 12.9	102.4 ± 15.7	0.0726	97.9 ± 15.6	94.3 ± 14.9	100.9 ± 15.8	0.1708
HbA1c % value (mean ± SD)	6.0 ± 0.3	5.8 ± 0.2	6.1 ± 0.2	0.0018	5.9 ± 0.3	5.8 ± 0.2	6.0 ± 0.2	0.0062
GDF-15 (pg/mL) (mean ± SD)	360.6 ± 117.9	377.3 ± 143.2	344.6 ± 87.7	0.3583	359.4 ± 118.9	396.1 ± 154.8	330.0 ± 71.3	0.0978
HOMA-IR	9.8 ± 7.2	9.5 ± 7.0	10.0 ± 7.6	0.8463	9.3 ± 7.4	9.9 ± 8.2	8.9 ± 7.0	0.7097
Total estimated carbs (g) (mean ± SD)	158.2 ± 68.4	153.4 ± 58.9	163 ± 77.7	0.6401	151.6 ± 58.6	144.3 ± 46.6	157.4 ± 67.2	0.5149
Total estimated added sugars (g) (mean ± SD)	48.2 ± 48.1	48.2 ± 32.7	48.2 ± 60.5	0.9983	41.9 ± 30.9	46.5 ± 31.4	38.3 ± 30.9	0.4384

Note: *p*-value from Fisher’s exact test for categorical variables and *t*-test for continuous variables.

**Table 2:** Changes from baseline to the follow-up at 12 weeks among the participants with a follow-up at 12 weeks. Unsweetened beverage group (USB); artificially sweetened beverage group (ASB).

	Overall (N = 36)	USB (N = 16)	ASB (N = 20)	*p*-Value ^(1)^
Have you noticed any changes in hunger? (N,%)				0.6665
Less hungry	23 (63.9%)	9 (56.3%)	14 (70.0%)	
No change	10 (27.8%)	5 (31.3%)	5 (25.0%)	
More hungry	3 (8.3%)	2 (12.5%)	1 (5.0%)	
Have you noticed any changes in your portion sizes? (N,%)				0.3204
Smaller	15 (41.7%)	5 (31.3%)	10 (50.0%)	
Same	21 (58.3%)	11 (68.8%)	10 (50.0%)	
Have you noticed any changes in snack intake? (N,%)				0.7343
Less snacks	22 (61.1%)	9 (56.3%)	13 (65.0%)	
No change	14 (38.9%)	7 (43.8%)	7 (35.0%)	
Change in BMI (mean ± SD)	−0.37 ± 1.54	−0.55 ± 1.49	−0.23 ± 1.60	0.5390
Change in BMI 95% percentile (mean ± SD)	−3.02 ± 5.83	−3.94 ± 5.78	−2.28 ± 5.91	0.4037
Change in BMI z scores (mean ± SD)	−0.10 ± 0.19	−0.12 ± 0.19	−0.09 ± 0.21	0.6273
Change in fasting glucose (mg/dL) (mean ± SD)	1.14 ± 21.40	−4.06 ± 18.83	5.30 ± 22.85	0.1962
Change in HbA1c (mean ± SD)	−0.12 ± 0.26	−0.12 ± 0.25	−0.13 ± 0.28	0.9445
Change in GDF-15 (mean ± SD) ^(2)^	25.38 ± 75.05	33.40 ± 58.34	19.77 ± 85.87	0.6099
Change in HOMA-IR (mean ± SD) ^(3)^	−0.22 ± 10.16	−4.13 ± 9.68	1.84 ± 10.04	0.1354
Change in total estimated carbs (g) (mean ± SD)	−23.60 ± 70.97	−22.10 ± 68.10	−24.80 ± 74.92	0.9092
Change in total estimated added sugars (mean ± SD)	−19.50 ± 33.94	−22.20 ± 39.98	−17.40 ± 29.16	0.6800
Percentage of metformin intake (mean ± SD)	70.00 ± 31.05	70.00 ± 36.97	70.00 ± 26.12	1.0000

^(1)^ *p*-value from Fisher’s exact test for categorical variables and *t* test for continuous variables. ^(2)^ N in GDF-15: total = 34 (USB arm = 14, ASB arm = 20). ^(3)^ N in HOMA-IR: total = 29 (USB arm = 10, ASB arm = 19).

**Table 3:** Binary categorization of changes from baseline to the follow-up at 12 weeks among the participants with a follow-up at 12 weeks. Unsweetened beverage group (USB); artificially sweetened beverage group (ASB).

	Overall		USB		ASB		*p*-Value
	N	%	N	%	N	%	
Decrease in BMI	19	52.8	9	56.3	10	50.0	0.7486
Yes							
No	17	47.2	7	43.8	10	50.0	
Decrease in BMI 95% percentile	23	63.9	10	62.5	13	65.0	1.0000
Yes							
No	13	36.1	6	37.5	7	35.0	
Decrease in BMI z-score	24	66.7	10	62.5	14	70.0	0.7295
Yes							
No	12	33.3	6	37.5	6	30.0	
Decrease in HbA1c	24	66.7	10	62.5	14	70.0	0.7295
Yes							
No	12	33.3	6	37.5	6	30.0	
Increase in GDF-15	2	5.6	2	12.5	0	0	0.1625
Missing							
Yes	16	44.4	9	56.3	7	35.0	
No	18	50.0	5	31.3	13	65.0	
Decrease in HOMA-IR	7	19.4	6	37.5	1	5.0	0.0084
Missing							
Yes	16	44.4	9	56.3	7	35.0	
No	13	36.1	1	6.3	12	60.0	

NOTE: missing values were removed for the bivariate analysis. *p*-value from Fisher’s exact test.

## Data Availability

The raw data supporting the conclusions of this article will be made available by the authors, without undue reservation.

## Supplementary Materials

The following supporting information can be downloaded at: https://www.mdpi.com/article/10.3390/nu17050797/s1, Supplementary S1: Beverage Frequency Assessment Questionnaire; Supplementary S2: Study Design Graphic.
