# Supplementary material for: The Impact of Artificially Sweetened Drinks on Metformin Efficacy
Source: Nutrients. 2025 Feb 26;17(5):797. doi: 10.3390/nu17050797 (PMC11901812; doi:10.3390/nu17050797)
Supplement: Supplementary file 1 [file nutrients-17-00797-s001.zip › nutrients-3405973-supplementary.pdf]

Supplementary Materials 1

UF | Department of Pediatrics
UNIVERSITY *of* FLORIDA

Participant ID: ____________

Date: ________

Beverage Frequency Assessment Questionnaire

Instructions:
In the past week, please indicate your response for each beverage type by checking the box for how often you consumed each type of beverage.
For example, if you drank 1 cup of water 2 times per day this week, mark the "2 times per day" column in the "water" row.

	Type of Beverage	Never or less than 1 time per week	1 time per week	2-3 times per week	1 time per day	2 times per day	3+ times per day
DRINKS WITH SUGAR	Regular soda or pop (Coke, Pepsi, 7-Up, Sprite, etc.)	☐	☐	☐	☐	☐	☐
	100% fruit or vegetable juice (orange juice, apple juice, etc.)	☐	☐	☐	☐	☐	☐
	Sweetened fruit drinks (lemonade, iced tea, fruit punch/cocktail, Kool Aid, Capri Sun, etc.)	☐	☐	☐	☐	☐	☐
	Sweetened waters or vitamin waters (with sugars)	☐	☐	☐	☐	☐	☐
	Sport and energy drinks with sugar (Gatorade, Powerade, Red Bull, Monster, etc.)	☐	☐	☐	☐	☐	☐
	Chocolate milk, sweetened milk alternatives or other flavored milk	☐	☐	☐	☐	☐	☐
	Regular coffee or tea with sugar or sweet creamer	☐	☐	☐	☐	☐	☐
	Specialty coffee with sugar (lattes, mochas, Frappuccino, macchiatos, etc.	☐	☐	☐	☐	☐	☐
	Meal replacements (shakes, protein drinks) with sugar	☐	☐	☐	☐	☐	☐
	Beer, cider or coolers	☐	☐	☐	☐	☐	☐
	Wine (red or white)	☐	☐	☐	☐	☐	☐
	Hard alcohol with mix, cocktails that have calories	☐	☐	☐	☐	☐	☐
	Other drinks sweetened with sugar: ____________	☐	☐	☐	☐	☐	☐

	Type of Beverage	Never or less than 1 time per week	1 time per week	2-3 times per week	1 time per day	2 times per day	3+ times per day
DIET DRINKS (WITH ARTIFICIAL SWEETENERS)	Diet soda or pop (Coke Zero, Diet Pepsi, etc.)	☐	☐	☐	☐	☐	☐
	Sugar-free drinks that are NOT pop (artificially sweetened Crystal Light, Mio, flavored waters with no calories, etc.)	☐	☐	☐	☐	☐	☐
	Sport and energy drinks with no calories (artificially sweetened, no sugar)	☐	☐	☐	☐	☐	☐
	Regular coffee or tea sweetened with artificial sweeteners (with/without plain cream, no sugar)	☐	☐	☐	☐	☐	☐
	Meal replacements (shakes, protein drinks) with artificial sweeteners	☐	☐	☐	☐	☐	☐
	Hard alcohol with diet mixes	☐	☐	☐	☐	☐	☐
	Other drinks sweetened with artificial sweeteners: ____________	☐	☐	☐	☐	☐	☐
UNSWEETENED DRINKS (NO SUGAR, NO ARTIFICIAL FLAVORS)	Water	☐	☐	☐	☐	☐	☐
	Flavored unsweetened water/sparkling water (Polar, La Croix, Dasani, Bubly, Waterloo, AHA, (Hint, JUST water, Rethink, Wonder+Well, True Lemon FRUIT INFUSIONS)	☐	☐	☐	☐	☐	☐
	White milk (whole milk, 2% milk, 1% milk and fat-free milk) or unsweetened milk alternatives (soy or nut milk, etc.)	☐	☐	☐	☐	☐	☐
	Regular coffee or tea with no sugar or artificial sweeteners (with/without plain cream)	☐	☐	☐	☐	☐	☐
	Other unsweetened drinks: ____________	☐	☐	☐	☐	☐	☐

***Artificial sweeteners include:** aspartame (Equal, NutraSweet), saccharin (Sweet'N'Low, Sugar Twin), sucralose (Splenda), acesulfame potassium (Sunett), stevia (Truvia, PureVia, SweetLeaf), sugar alcohols [erythritol, glycerol, isomalt, lactitol, maltitol, mannitol, sorbitol, xylitol and hydrogenated starch hydrolysates (HSH)), advantame, neotame (Newtame)]

PS161311-11/22/21

Supplementary Materials 2

Study Design

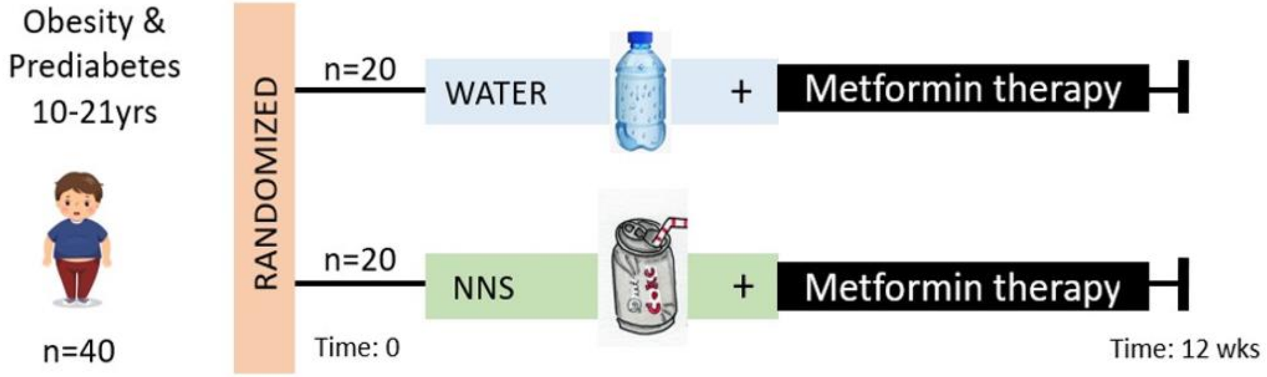

Randomized to:
Counseling on Beverage Intake
+
Metformin therapy

Data collection measures & time point	
**Measure**	**Time point**
Height, weight, BMI	Baseline, 12 weeks
GDF-15 ELISA blood collection	Baseline, 12 weeks
A1C, fasting BG, fasting insulin	Baseline, 12 weeks
Beverage intake*	Every 2 weeks
24-hr dietary recall	Baseline, 12 weeks
Hunger assessment	Baseline, 12 weeks
